# The Role of Ultra-High-Frequency Ultrasound in Pyoderma Gangrenosum: New Insights in Pathophysiology and Diagnosis

**DOI:** 10.3390/diagnostics13172802

**Published:** 2023-08-30

**Authors:** Giammarco Granieri, Alessandra Michelucci, Flavia Manzo Margiotta, Bianca Cei, Saverio Vitali, Marco Romanelli, Valentina Dini

**Affiliations:** 1 Department of Dermatology, University of Pisa, 56126 Pisa, Italy; giammarcogranieri@gmail.com (G.G.); manzomargiottaflavia@gmail.com (F.M.M.); ceibianca95@gmail.com (B.C.); marco.romanelli@unipi.it (M.R.); valentina.dini@unipi.it (V.D.); 2 Diagnostic and Interventional Radiology, University Hospital of Pisa, 56126 Pisa, Italy; vitalisaverio@gmail.com

**Keywords:** pyoderma gangrenosum, ultra-high-frequency ultrasound, UHFUS, PG

## Abstract

Pyoderma gangrenosum (PG) is a neutrophilic dermatological disease, whose pathogenesis is still poorly clarified. Because of the lack of validated criteria for diagnosis and response, PG treatment is still challenging and should be differentiated in the inflammatory and non-inflammatory phases. Our study aimed to provide a new semi-quantitative approach for PG diagnosis and monitoring, identifying ultra-high-frequency ultrasound (UHFUS) early biomarkers associated with the transition between the two phases. We enrolled 13 patients affected by painful PG lesions evaluated during the inflammatory phase (T0) and during the non-inflammatory phase (T1): pain was measured by the Visual Analogue Scale (VAS); clinical features were recorded through digital photography; epidermis and dermis ultrasound (US) characteristics were evaluated by UHFUS examination with a 70 MHz probe (Vevo MD^®^ FUJIFILM VisualSonics). In T1 UHFUS examination, the presence of hyperechoic oval structures was lower compared to T0 (*p* value < 0.05). An hyperechogenic structure within the oval structure, suggestive of a hair tract, was evident in T0 and absent in T1 (*p* value < 0.05). In T0, blood vessels appear as U-shaped and V-shaped anechoic structures with a predominance of U-shaped vessels (*p* value < 0.05) compared to the more regular distribution found in T1. Finding early biomarkers of the transition from the inflammatory to the non-inflammatory phase could provide new insight in terms of therapeutic decision making and response monitoring. The differences found by this study suggest a potential use of UHFUS for the development of an objective standardized staging method. Further investigations will be necessary to confirm our preliminary results, thus providing a turning point in PG early detection, differential diagnosis and treatment monitoring.

## 1. Introduction

Pyoderma gangrenosum (PG) is a neutrophilic dermatosis of unknown etiology and uncertain epidemiology due to its challenging diagnosis. Its estimated prevalence is approximately 58 cases per million people, with an incidence of approximately 6 cases per million [1,2].

The average age of onset is 59 years with a higher prevalence in women, even if some studies suggest that the higher incidence occurs between 20 and 50 years old, without gender differences [2,3]. The mortality rate associated with PG ranges from 16% to 27% [4].

Furthermore, approximately 50% of PG patients are affected by another immune disease, although some studies report a slightly lower frequency [5]. Notably, PG is the second most common cutaneous manifestation of inflammatory bowel disease (IBD) [4].

The epidemiology of PG varies depending on the clinical phenotypes, which includes ulcerative, pustular, bullous and vegetative forms. Post-surgical PG is considered a specific entity that can represent a climax of pathergy phenomenon. The ulcerative phenotype is the most prevalent form, primarily affecting the lower limbs. PG is frequently associated with IBD, rheumatoid arthritis (RA), seronegative arthritis, monoclonal gammopathy, and hematological malignancies [5,6,7].

Ulcerative PG is characterized by rapidly expanding painful ulcers, often exhibiting a distinctive lilac ring at the edge [4].

All phenotypes of PG present an inflammatory phase characterized by high levels of pain. Therefore, the primary step of therapy is to block the inflammatory process, with immunosuppressive/immunomodulatory drugs and appropriate wound care, based on PG TIME [5,8]. Following the proper treatment, this phase moved toward a non-inflammatory phase that allows wound re-epithelialization [9,10,11].

Because of the chronic and relapsing course of PG, finding early biomarkers of this transition could be useful in terms of therapeutic decision making and response monitoring [5].

Currently, PG remains a diagnosis of exclusion as there are no validated and specific clinical, instrumental, or serological markers. Histological examination is non-specific and can vary depending on the sample site and stage of the lesion [12].

As a result, diagnostic tools, such as the PARACELSUS score, have been proposed, incorporating major, minor and additional criteria based on clinical, histological and therapeutic features of the ulcer [5,13]. Only one study is in the literature regarding the use of UltraSonography (US) with a linear probe up to 18 MHz in PG. This examination performed at the level of a painful nodule from PG showed a well-defined hypoechogenic structure at the subepidermal level, which continued with a hypoechogenic, heterogeneous, irregular area that disposed in sections reaching a destructured hypodermis. Doppler color modality showed increased local vascularity. After one week of treatment, a reduction in local vascularity and lesion dimensions was identified. There are no applications of ultra-high-frequency ultrasound (UHFUS) in PG lesions in the literature [14].

As technology continues to advance and research progresses, UHFUS is poised to become an indispensable tool in dermatology, revolutionizing the way we understand and address various skin conditions. Furthermore, the advancement in imaging techniques, specifically UHFUS, could offer distinct advantages over conventional diagnostic methods, as it is a non-invasive procedure that delivers a spatial resolution in the order of 30 μm. This level of resolution allows for the visualization of skin structures with unprecedented clarity, comparable to what is achievable through histological examination. UHFUS provides clinicians and researchers with a powerful tool to delve deep into the skin’s microanatomy, revealing intricate details that were previously inaccessible without invasive procedures. The technique captures comprehensive images of various skin components, such as the dermo-epidermal junction, hair follicles, pilo-erector muscles, and blood vessels [15,16,17,18,19,20,21]. The distinct patterns observed through UHFUS imaging could assist in the early detection of PG, enabling timely intervention and management. Moreover, UHFUS serves as an essential tool in monitoring the progression of PG and evaluating the efficacy of treatment. By conducting longitudinal UHFUS assessments, clinicians could track changes in the affected skin over time, observing how the lesions evolve and respond to therapeutic interventions. This real-time feedback enhances the precision of treatment plans, as adjustments can be made based on objective imaging data, leading to more personalized and effective patient care.

The non-invasive nature of UHFUS also addresses concerns related to the risk of pathergy phenomenon often associated with invasive procedures such as skin biopsies. This reduction in invasiveness not only ensures patient comfort and safety but also encourages more frequent monitoring, facilitating a proactive approach to PG management.

Despite its tremendous potential, UHFUS is still a developing field, and further research is warranted to fully understand its capabilities in PG diagnosis and management. Collaborative efforts between dermatologists, imaging specialists, and researchers will be essential to optimize UHFUS protocols and establish standardized criteria for PG diagnosis based on imaging findings.

Its non-invasive nature, coupled with remarkable spatial resolution, could provide valuable insights for accurate diagnosis, treatment planning, and monitoring of PG.

The aim of this study was to identify UHFUS biomarkers associated with the inflammatory and non-inflammatory phases of different phenotypes of PG.

## 2. Materials and Methods

We enrolled 13 patients affected by painful PG lesions (Visual Analogue Scale (VAS) > 8): 11/13 presented an ulcerative phenotype, 2/13 had a pustular phenotype and 5/13 showed multiple PG lesions in different body areas. PG diagnosis was performed by a PARACELSUS score ≥ 10 and a skin biopsy with histological findings revealing intense neutrophilic infiltrate [13]. 

All patients were evaluated during the inflammatory phase (T0) and during the non-inflammatory phase (T1). T0 is defined by VAS > 7 and rapid growth and development of new lesions. In ulcerative PG, this phase was also characterized by the presence of a lilac ring, severe exudate and necrosis on the wound bed. In the pustular phenotype, the occurrence of new pustular satellite lesions and the increase in perilesional skin erythema suggested the presence of the inflammatory phase.

T1 was defined by VAS < 2, the absence of a lilac ring, wound bed necrosis and new satellite lesions for more than 4 weeks.

PG management was performed with a combination of local and systemic therapy, according to evidence and expert opinions presented in the literature [8,22,23].

**Figure 1:**
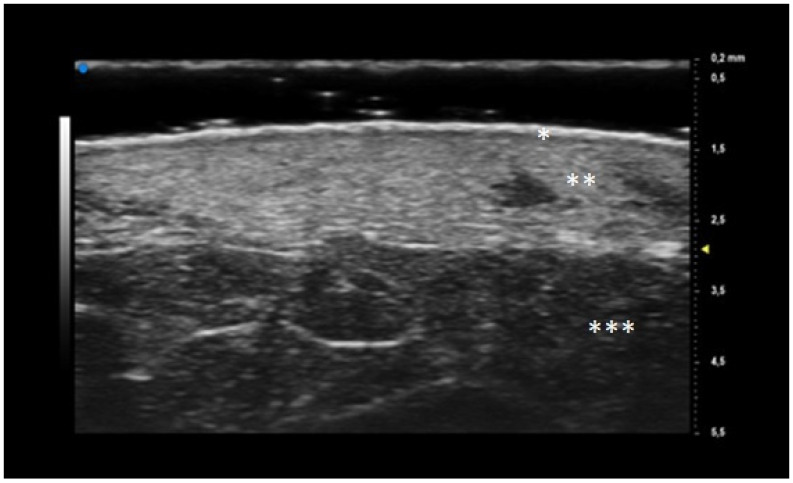
UHFUS features of healthy skin: epidermis (*), dermis (**) and hypodermis (***).

A comprehensive patient’s assessment was performed at T0 and T1. The clinical investigation was performed by a dermatologist expert in pyoderma gangrenosum which collected a photographic record of the patient and assessed clinical disease parameters such as pain, measured with VAS. UHFUS investigation was performed by a dermatologist expert in UHFUS blinded from the clinical diagnosis by three UHFUS clips with a 70 MHz linear probe (Vevo MD^®^ FUJIFILM VisualSonics, Toronto, Ontario, Canada), in B-MODE. The use of UHFUS with a 70 MHz probe allows to examine the more superficial cutaneous and adnexal features with a spatial resolution in the order of 30 μm (Figure 1). A correlation between digital photograph and US clip was performed: two regions of interest (ROI) at the wound bed and edge were provided for the ulcerative phenotype, while one ROI was provided for the pustular phenotype. The probe was placed perpendicular to the lesion and a large amount of gel was used to maintain the adequate distance from the skin surface. The US parameters (such as gains, depth, time gain control, focus) were optimized during the examinations. A qualitative-quantitative analysis of the US features was performed by 2 dermatologists experienced in UHFUS imaging.

The UHFUS characteristics evaluated were:

Epidermis and dermis US morphology

Vessel’s morphology

Dermal oedema as dermal hypoechogenicity, classified in three degrees (mild, moderate, severe)

Aliasing as the presence of image background noise, classified in three degrees (mild, moderate, severe).

Categorical data were described with absolute and relative (%) frequency, continuous data were summarized with mean and standard deviation. Fisher test was performed to compare UHFUS results obtained by the population evaluated in T0 and T1. Significance was set at <0.05 and all analyses were carried out by SPSS v. 28 technology.

## 3. Results

Our population consisted of 13 patients (11 females and 2 males) with a mean age of 60.38 (17.4) years, a mean disease duration of 5 years and a mean BMI of 30.55 (5.7). The clinical and anamnestic features of the population are reported in Table 1, while the results obtained by UHFUS examination are presented in Table 2. The mean time needed to switch from the T0 phase to the T1 phase was 2 months.

Oval hyperechoic structures were identified in the reticular and papillary dermis during the initial T0 UHFUS examination. These structures were surrounded by a consistent, homogeneous hypoechoic background. Notably, this peculiar arrangement was observed in both the pustular and ulcerative phenotypes, indicating a common underlying feature.

As the examination progressed towards the inflamed edge, these oval structures exhibited a noticeable increase in prevalence, reinforcing their association with the inflammatory process. 

**Figure 2:**
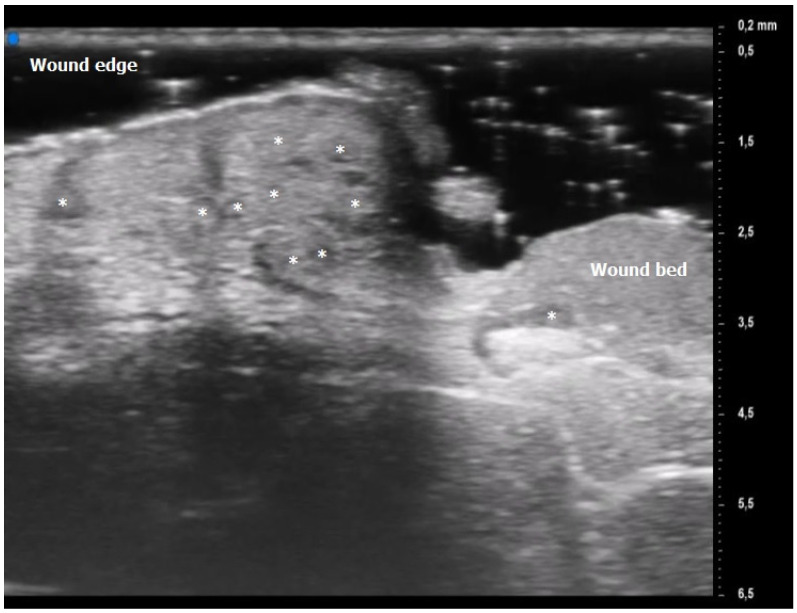
Pyoderma Gangrenosum (PG) Ultra-High Frequency UltraSound (UHFUS) features during the inflammatory phase (T0): oval hyperechoic structures (*) surrounded by hypoechoic borders predominantly located at the ulcer edge.

A statistically significant decrease in the presence of these hyperechoic oval structures was observed during the subsequent T1 UHFUS examination. This reduction in prevalence (*p* value < 0.05) underscores the dynamic nature of these structures and their potential correlation with the progression of the inflammatory response (Figure 2).

**Figure 3:**
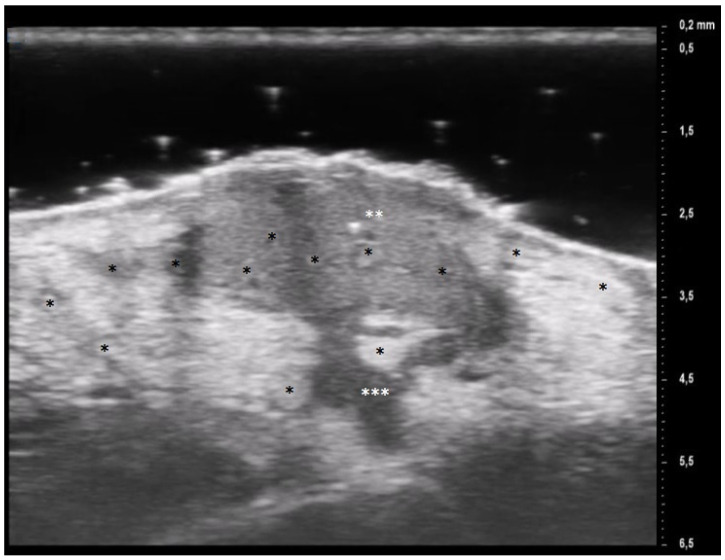
PG pustular lesion: hyperecoic oval structures (*), hair tract (**), hypoechoic U-shaped and V-shaped vessels (***).

Additionally, a distinct finding emerged during the T0 UHFUS examination—an internal hyperechogenic structure within the oval entities. This internal structure, suggestive of a hair tract, was consistently identified during the initial examination (T0) but was notably absent in the follow-up examination (T1) (*p* value < 0.05) (Figure 3).

During the initial T0 examination, blood vessels appeared as U-shaped and V-shaped anechoic structures within the observed lesion. Notably, these vessels exhibited a distinct orientation, with their concavity consistently facing the center of the lesion. Moreover, a statistically significant predominance of U-shaped vessels was detected in T0 (*p* value < 0.05).

Transitioning to the subsequent T1 examination, the blood vessels appeared to adopt a more regular distribution within the lesion. This shift was accompanied by a decline in both U-shaped and V-shaped vessels, indicative of an evolving vascular pattern over the course of progression. 

The phenomenon of aliasing, characterized by the distortion of signals in ultrasound imaging, was notably pronounced (moderate to severe) during the initial assessment at T0. As the examination progressed to the subsequent time point, T1, a discernible improvement was observed in the degree of aliasing. 

## 4. Discussion

Pyoderma gangrenosum is a dermatological disease with a great socio-economic burden on patients’ quality of life. Its chronic and relapsing nature necessitates the need for an early recognition and prompt management of a new disease flare. Goldust et al. assessed that the main research gap in the PG field is related to the identification of diagnostic biomarkers and standardized staging methods [24,25,26,27].

The substantial therapeutic differences between inflammatory and non-inflammatory phases require imaging tools and quantitative parameters to guide and support treatment choice. Moreover, recognition of early inflammatory signs would be crucial to avoid the pathergy phenomenon occurrence. 

The distinction between the inflammatory and non-inflammatory phases of PG is essential for the choice of the correct therapeutic approach, either topical or systemic. Surgical debridement, as well as other traumatic treatments, can be carried out only during the non-inflammatory phase, because of the pathergy phenomenon. On the other hand, the choice of frequency and dose administration of immunosuppressive and immunomodulatory drugs is guided by the disease phase. During the active and acute phases of the disease, a more aggressive treatment approach may be necessary. Higher doses and more frequent administrations of immunosuppressive drugs may be prescribed to quickly suppress the overactive immune response and control the progression of the condition. Close monitoring by medical professionals is essential during this phase to assess the response to treatment and to ensure the patient’s safety. Conversely, during the remission or maintenance phase of the disease, the treatment strategy may shift towards a more conservative approach. Lower doses or less frequent administrations of immunosuppressive drugs might be prescribed to maintain the disease in a controlled state and prevent flare-ups. The objective in this phase is to find a balance between regulating the immune system and avoiding excessive risks linked to prolonged immunosuppression. To date, the assessment of the inflammatory phase in PG is performed exclusively by clinical examination of the lesions: an intensified pain experienced by the patient, an increase in the number of lesions, and the presence of lilac ring are clinical parameters suggestive of a transition to the inflammatory phase. The presence of comorbidities or wound superinfections may mask or mimic an inflammatory phase, thus making its detection more difficult.

It is therefore a current challenge to identify early and more objective biomarkers, detectable even before clinical parameters, suggestive of the transition from the inflammatory phase to the non-inflammatory one, to guide therapeutic choice.

In our population, the mean age of PG onset was 55 years old, with a higher prevalence in women (M/F ratio of 0.18) in agreement with evidence presented in the literature [2]. The most frequently involved site was the lower limbs (84.6%), and the most observed comorbidities were HS (61.4%), hematologic malignancies (15.4%) and IBD (7.7%) as Maverakis et al. had already reported [5]. Moreover, in our population PG was often associated with psoriasis: 30.8% of patients presented a positive family history while 23.1% were affected by psoriasis. These results could be explained by the common proinflammatory pathways shared between the two diseases [4].

In this study, for the first time in the literature, clinical parameters were correlated with UHFUS findings obtained with a 70 MHz probe. Only one case report described the use of US (18 MHz) in PG, revealing the presence of heterogeneous and irregular hypoechogenic dermis that changes after systemic corticosteroid therapy with an increased dermal echogenicity [14].

**Figure 4:**
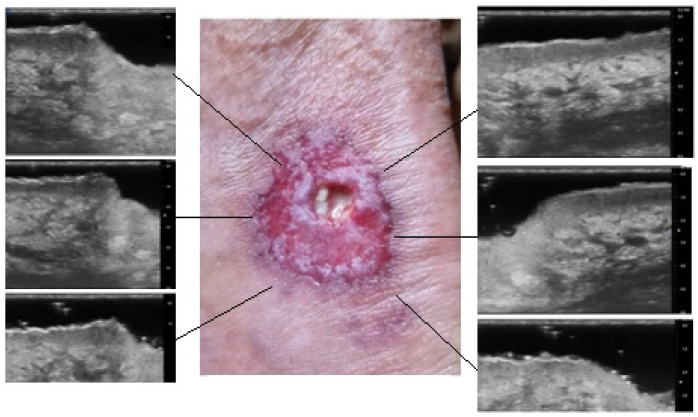
Patient affected by ulcerative Pyoderma Gangrenosum (PG) of the calf during the inflammatory phase: clinical aspect of the wound and its Ultra-High Frequency UltraSound (UHFUS) correlates.

**Figure 5:**
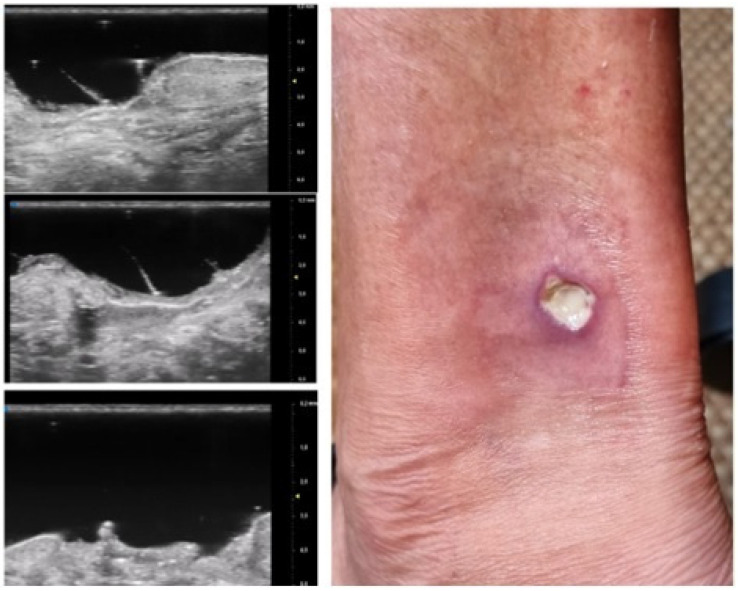
Patient affected by ulcerative Pyoderma Gangrenosum (PG) of the calf during the non-inflammatory phase: clinical aspect of the wound and its Ultra-High Frequency UltraSound (UHFUS) correlates.

The results obtained by our investigation revealed some UHFUS differences between the inflammatory and non-inflammatory phases. At T0, oval hyperechoic structures, that statistically significantly decreased in T1, were identified in the papillary and reticular dermis (*p*-value < 0.05) (Figure 4 and Figure 5).

**Figure 6:**
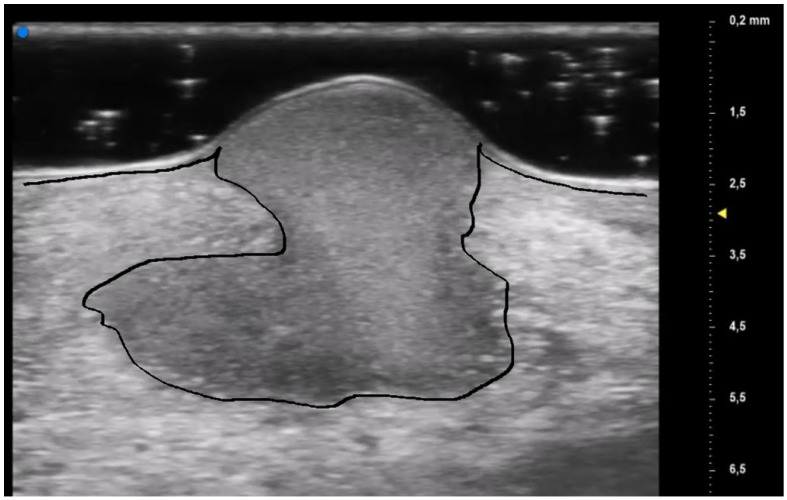
Early PG lesion: the purulent material (included by the black line) is well demarcated and undermined from the surrounding dermis giving a wave-like appearance, the so-called “tsunami sign”.

These US findings spared the epidermis and were well demarcated from the surrounding dermis, thus suggesting a hypothesis of “dermal destruction” that we called “tsunami sign” because of the presence of a US image resembling a wave breaking towards the center of the lesion (Figure 6). In T0, hyperechoic oval structures were mainly located at the level of the lesion edges, near V-shaped and U-shaped blood vessels, where the inflammatory response presented a higher activity. Particularly, in T0 an increased expression of U-shaped vessels compared to V-shaped vessels was detectable. In contrast, T1 was characterized by a more uniform vascularization with a significant reduction in U-shaped vessels (*p*-value < 0.05). These UHFUS findings could be explained by the increased dermal oedema in T0, which resulted in surrounding connective tissue compression and morphological blood vessels changes.

In addition, we identified an augmented echogenicity of the dermis in T0 compared to T1, probably due to increased dermal oedema during the inflammatory phase.

On T0 B-MODE examination we also noticed an enhancement of background noise (called “aliasing”) in proximity of V-shaped and U-shaped vessels and oval formations. While the vascular signal in C-MODE could be non-specific, the B-MODE signal allowed us to identify a background noise related to increased reflection phenomena, caused by the presence of corpuscular elements, that, due to vasodilation and stasis, are piled in the blood vessels. The aliasing signal was more easily detectable at the wound edges, probably because of the increased vasodilatation responsible for the lilac ring formation, and it was reduced in T1.

The UHFUS characteristics found in T0 decreased in number and density in T1, but did not disappear. This result could be explained by two theories. First, the permanence of some UHFUS features could be suggestive of a new potential inflammatory flair, thus justifying the need for immunomodulatory maintenance therapy even in the non-inflammatory phase. In addition, the persistence of oval formations/micro-abscesses and V-shaped vessels in the non-inflammatory phase promoted the hypothesis that these UHFUS patterns were pathognomonic signs of PG.

**Figure 7:**
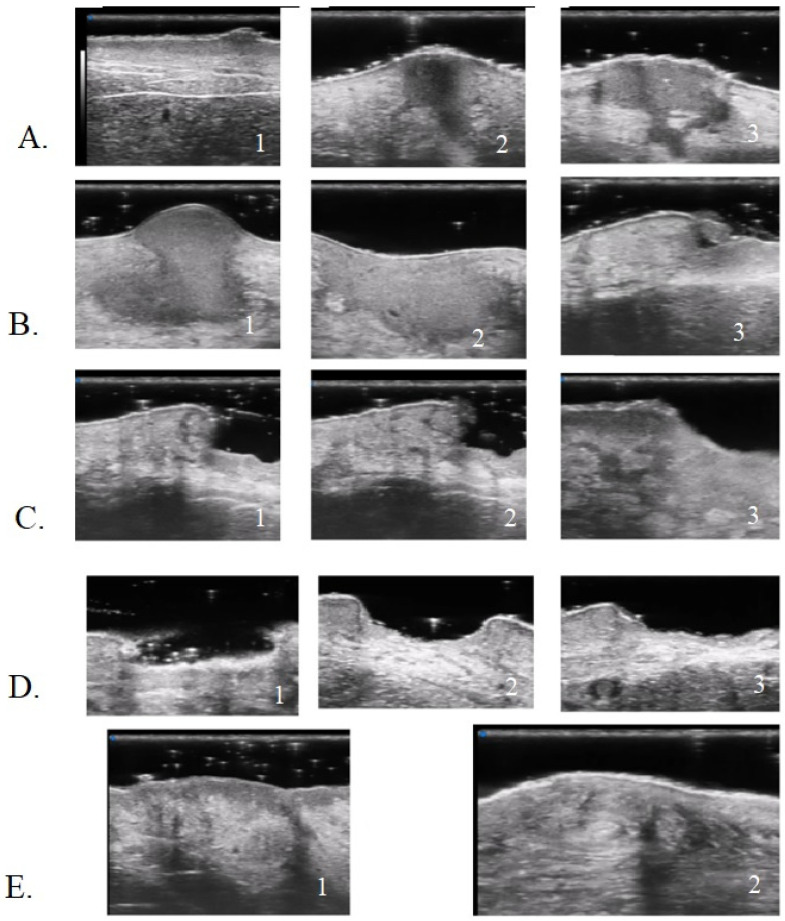
UHFUS PG features: early PG pustule with hair tract (**A1-2-3**); undermined purulent material with the “tsunami sign” formation (**B1-2-3**); PG evolution toward ulceration (**C1-2-3**); PG lesion during non-inflammatory phase (**D1-2-3**); healed PG lesion (**E1-2**).

Moreover, the presence of similar UHFUS findings between the pustular and ulcerative phenotypes in T0, suggested the idea, not yet confirmed in the literature, that the pustular phenotype was an early stage of the ulcerative one, in agreement with the assumption of Powell et al. this hypothesis could be supported by our population’s anamnestic data that reveal a clinical onset with confluent or non-confluent pustules in 5 patients. (Figure 7) [12].

UHFUS examination showed the presence of a hair track within the oval hyperechoic structures in T0 and their disappearance in T1 (*p*-value < 0.05). This finding, as well as the increased U-shaped and V-shaped vessels in T0, could also support the idea that the pilosebaceous unit could play a pivotal role in PG pathogenesis.

Wang et al. demonstrated that healed PG scars presented a complete loss of the pilosebaceous unit. Perilesional ulcer skin biopsy revealed a predominance of lymphocytes T polyclonal against dermal and follicular antigens. Moreover, early PG papules revealed higher expression of genes coding for chemokines that attract lymphocytes T, thus causing perivascular and peri pilosebaceous T cell infiltrates [28,29].

In addition, Marzano et al. identified increased activation of adaptive immunity toward hair follicle Ag, as histological analysis performed at the ulcer edge demonstrated the presence of clonally expanded T cells, which could indicate an antigen-driven phenomenon [5].

Hurwitz et al. conducted a comparative study between the different stages of PG: nine skin biopsies were performed on evolving, active, regressing or resolved PG lesions.

The early papule-pustules presented deep folliculitis involving the pilosebaceous unit, with neutrophils located in and surrounding the infundibulum that showed signs of rupture or perforation. 

Ulcerated and inflamed lesions were characterized by a massive papillary dermal edema with epidermal neutrophilic abscesses that contributed to the peripheral violaceous undermined edges.

The healing phase presented infiltrates of lymphocytes and histiocytes, while completely healed lesions showed marked signs of fibroplasia [30].

However, a skin biopsy is an invasive exam that depends on the sample site and the lesion’s stage. UHFUS (70 MHz) with a spatial resolution in the order of 30 microns permitted us to perform a kind of in vivo histological examination. This non-invasive and repeatable exam allowed real-time monitoring of PG lesions, and finding a correlation between UHFUS morphological structures and histological features would represent a breakthrough in PG diagnosis and treatment monitoring.

The histological findings reported by Hurwitz et al. were comparable with UHFUS ones: the “hair tract” as a sign of pilosebaceous unit involvement; the “dermal hypoecogenity” as a mark of oedema; the “oval hyperechogenic structure” as neutrophilic microabscesses and the “increasing in dermal echogenicity” as increased fibroplasia in the healing phase. 

Moreover, our study provides new insight into PG pathogenesis comprehension: dermal abscesses and their subsequent ulceration could derive from an immune response directed toward exposed follicular antigens [29]. 

## 5. Conclusions

Our study aimed to provide a new semi-quantitative approach for PG diagnosis and monitoring.

The use of US, particularly UHFUS examination, has demonstrated its potential to revolutionize the field of dermatology by providing valuable insights into the development of an objective and standardized staging method for various skin disorders [31,32,33,34]. In this context, our study represents the first experience with UHFUS in patients affected by PG lesions presented in the literature. Notably, one of the significant advantages of UHFUS examination is its ability to facilitate early diagnosis without the associated risks of pathergy phenomenon induced by invasive procedures such as skin biopsies. From the preliminary results obtained with this study, the use of UHFUS examination has shown promising potential in standardizing staging methods and enabling early diagnosis of various skin conditions, including PG.

However, there are some limitations in our study that need to be analyzed and overcome with further investigations in order to understand the full potential of UHFUS as an invaluable tool in PG diagnosis and patient care.

The foremost limitation lies in the small sample size, which might have impacted the generalizability of the findings. In future studies, it will be necessary to include larger and more different patient cohorts to ensure more comprehensive results. Moreover, it would be interesting to perform a quantitative assessment of the vascular signal for a better US characterization of the lesions.

Additionally, the variation in treatments received by the patients in this study poses another challenge. Different treatment approaches might have influenced the presentation of the pustules or ulcerative lesions, which could potentially confound the interpretation of the UHFUS results. To overcome this limitation, future investigations should include patients with more uniform treatment plans, allowing for a more focused evaluation of the imaging biomarkers.

To enhance the applicability of UHFUS in diagnosing PG and distinguish it from other chronic wounds, further research should include a broader spectrum of pustules or ulcerative lesions. By expanding the scope of investigation, we can identify specific imaging biomarkers that are uniquely associated with PG and aid in its differential diagnosis.

Lastly, while our study proposed a hypothesis regarding the pathogenesis of PG, it is crucial to validate this hypothesis through histological correlation. Histological examination of tissue samples from PG patients can provide valuable insights into the underlying mechanisms and confirm the accuracy of UHFUS findings.

## Figures and Tables

**Table 1:** Characteristics of the population (*n* = 13). Statistics: frequency (%).

Characteristics		Statistics
*Onset*	Confluent pustules	2 (15.4)
	Non-confluent pustules	3 (23.1)
	Nodule	5 (38.5)
	Blister	3 (23.1)
	Trauma	2 (15.4)
*Current phenotype*	Ulcerative	11 (84.6)
	Pustular	2 (15.4)
*Location*	Face	2 (15.4)
	Lower limbs	11 (84.6)
	Upper limbs	1 (7.7)
	Back	4 (30.8)
*Family history*	Hematological malignancies	1 (7.7)
	Solid tumors	7 (54.0)
	Rheumatoid arthritis	2 (15.4)
	Hypertension	6 (46.2)
	Diabetes	6 (46.2)
	Acne/hidradenitis suppurativa/pilonidal cyst	4 (30.8)
	Psoriasis	4 (30.8)
*Comorbidities*	Hematological malignancies	2 (15.4)
	Solid tumors	1 (7.7)
	Cardiovascular disease	7 (54.0)
	Diabetes	1 (7.7)
	IBD	1 (7.7)
	Acne/Hidradenitis suppurativa	8 (61.4)
	Psoriasis	3 (23.1)

**Table 2:** Ultra-High Frequency UltraSound (UHFUS) features of the population (*n* = 13). Statistics: frequency (%).

UHFUS Parameter		T0	T1	*p*-Value
Hyperechoic oval structures		12 (92.3)	4 (30.8)	0.001
Hair tract		7 (53.8)	0 (0)	0.005
V-shaped vessels		6 (46.2)	7 (53.8)	0.695
U-shaped vessels		12 (92.3)	5 (38.5)	0.004
Dermal hypercogenicity		0 (0)	2 (15.4)	0.8634
Dermal hypoechonicity	Mild	5 (38.5)	10 (69.2)	0.8634
	Moderate	6 (46.2)	1 (7.7)	
	severe	2 (15.4)	0 (0)	
Aliasing	Mild	3 (23.1)	9 (69.2)	0.636
	Moderate	6 (46.2)	1 (7.7)	
	Severe	5 (38.5)	0 (0)	

## Data Availability

The authors confirm that the data supporting the findings of this study are available from the corresponding author on request.
